# AI Images vs. Real Photographs: Investigating Visual Recognition and Perception

**DOI:** 10.3390/jemr18060061

**Published:** 2025-11-03

**Authors:** Veslava Osińska, Weronika Kortas, Adam Szalach, Marc Welter

**Affiliations:** 1 Institute of Information and Communication Research, Nicolaus Copernicus University, 87-100 Toruń, Poland; wkortas@umk.pl; 2 Institute of Informatics, College of Social and Media Culture, 87-100 Toruń, Poland; adam.szalach@aksim.edu.pl; 3 Inria Center at the University of Bordeaux, 33405 Talence Cedex, France; marc.welter@inria.fr; 4 LaBRI—Laboratoire Bordelais de Recherche en Informatique, 33405 Talence Cedex, France

**Keywords:** AI images, GAN graphics, eye tracking, visual perception, ChatGPT

## Abstract

Recently, the photorealism of generated images has improved noticeably due to the development of AI algorithms. These are high-resolution images of human faces and bodies, cats and dogs, vehicles, and other categories of objects that the untrained eye cannot distinguish from authentic photographs. The study assessed how people perceive 12 pictures generated by AI vs. 12 real photographs. Six main categories of stimuli were selected: architecture, art, faces, cars, landscapes, and pets. The visual perception of selected images was studied by means of eye tracking and gaze patterns as well as time characteristics, compared with consideration to the respondent groups’ gender and knowledge of AI graphics. After the experiment, the study participants analysed the pictures again in order to describe the reasons for their choice. The results show that AI images of pets and real photographs of architecture were the easiest to identify. The largest differences in visual perception are between men and women as well as between those experienced in digital graphics (including AI images) and the rest. Based on the analysis, several recommendations are suggested for AI developers and end-users.

## 1. Introduction

According to AI experts, Generative Adversarial Networks (GANs) are the most promising and popular application of AI to have emerged around the year 2020. The concept of GAN was developed by Ian Goodfellow and colleagues [1]. Since then, the issue of neural networks in image recognition and classification has broadened to include the creation of realistic graphics. A GAN produces extremely realistic effects by feeding in large amounts of image data available on the Internet and drawing on a competitive mechanism.

For instance, the popular FaceApp, which can make a face on a selected photo look younger or older or change the colour of the eyes, hairstyle, etc., has been launched to transform graphic material to suit the user’s wishes. A GAN also comes in handy for interior design, when different home decoration styles can be tested immediately by using a phone and an appropriate app. Such examples show that the technology has quickly moved from specialist research environments into everyday use, reaching a mass audience.

Recently, the photorealism of generated images has improved noticeably due to developments in GAN technology [2,3]. The relevant algorithm can produce high-resolution images of human faces and bodies, cats and dogs, vehicles, and other categories of objects that the untrained eye cannot distinguish from real photographs. On ThisPersonDoesNotExist, users see a human face as sharp as a photograph taken with a modern digital camera every time the page is refreshed. These faces look extremely realistic: facial features, skin texture, hair, and even skin defects are all clearly visible. Various art generators based on AI models such as Midjourney, DALL-E, Stable Diffusion, and Craiyon create such images via prompts [4].

The widespread accessibility of these tools has generated new social and scientific challenges. On the one hand, special tutorials with tips on distinguishing AI-generated images from real ones (hereinafter referred to as artefacts, i.e., graphical imperfections in digital imaging) are currently proliferating on the web. On the other hand, automatic AI detectors are being developed and made available online to identify generated images, such as AI or NOT. These examples highlight that the spread of GAN-generated content has become a technical, cultural, and perceptual issue.

Therefore, this study aims to examine how people perceive and distinguish AI-generated images from real ones, with a particular focus on the use of eye tracking methods. The motivation for this research stems from the growing presence of artificial images in everyday digital environments, which raises questions about trust, authenticity, and human cognitive processing. By combining AI detection tasks with ET technology, the study contributes to a deeper understanding of how individuals process visual information in this age of AI. This knowledge is relevant for scientific discussion as well as society, as it can support the development of tools and practices that help people navigate a world increasingly shaped by AI-generated content.

## 2. Latest Research

Early research into the relationship between humans and artificially generated images focused on distinguishing photorealistic graphics from natural photographs. Lyu and Farid [5] explored the limits of human perception in evaluating image realism, while Wang and Moulin [6] investigated how photorealistic and photographic images could be discriminated. Similarly, Lalonde and Efros [7] analysed image realism using colour compatibility as a diagnostic factor. These studies laid the foundation for understanding how individuals judge visual authenticity and also provided methodological approaches to measuring such judgments.

Parallel efforts were undertaken to develop forensic techniques to detect differences between natural and computer-generated images. For example, Khanna et al. [8] proposed methods for classifying images originating from scanners, digital cameras, and computer graphics, while Wen et al. [9] used colour models for identifying synthetic content. These early contributions combined technical detection with human perceptual judgments, underlining the difficulty in drawing clear distinctions between artificial and real visuals.

A significant turning point in this research area came with the increasing realism of AI-generated human faces. Mader et al. [10] showed that people’s ability to identify computer-generated portraits depends heavily on training and motivational incentives. More recent work has highlighted the limitations of human perception even further. Nightingale and Farid [11] demonstrated that AI-generated faces are indistinguishable from real ones and are often perceived as more trustworthy. Tucciarelli et al. [12] revealed that individuals may over-identify artificial faces as real. Psychological studies have supported these findings, with results suggesting that AI-generated faces can appear more convincingly human than actual photographs [13]. The phenomenon extends beyond facial perception: art research has shown that people sometimes prefer AI-generated artworks over real paintings [14].

Neuroscientific studies have provided complementary evidence by examining how the brain responds to artificial images. Moshel et al. [15] demonstrated that AI-generated faces can be reliably decoded from neural activity, suggesting that the brain encodes these artificial stimuli similarly to real ones. Pocol et al. [16] conducted a comprehensive survey on deepfakes, AI-generated humans, and related media, highlighting how far the field has advanced and underscoring the challenges posed by increasingly indistinguishable synthetic content.

A promising method for further exploring the perceptual mechanisms behind image recognition is eye tracking (ET). As Valuch et al. showed, gaze patterns can reveal priming effects while recognising natural scenes [17]. More broadly, ET provides a non-invasive method of capturing fine-grained indices of cognitive processes, as noted by Eckstein et al. [18]. ET, therefore, represents a valuable tool for complementing behavioural and neuroscientific research on synthetic image perception. However, despite its potential, there is still a lack of research that might directly combine AI detection tasks with ET techniques, leaving an important gap that this study aims to address.

Recently, studies have begun directly combining AI detection tasks with eye-tracking methods. For instance, Huang and colleagues [19] designed an experiment where participants distinguished between real and artificially generated faces (StyleGAN-3) while their gaze was recorded. The results showed that when the participants suspected that an image was artificial, their gaze became more detailed and focused on analysing specific features.

Similarly, de Winter’s group [20] presented participants with pairs of images (one real, one AI-generated) of buildings or vehicles and asked them to indicate which was artificial [20]. The study revealed a heuristic in which futuristic-looking images were more often perceived as AI-generated, indicating that the perception of “realness” depends not only on technical features but also on content and form.

In medical applications, AI images examined how radiologists differ in gaze behaviour when viewing real vs. AI-generated medical images, including first and last fixations and whether gaze was more dispersed or focused [21]. This suggests that AI-generated images can influence cognitive strategies in assessing authenticity.

Furthermore, works like Eye Tracking-Enhanced Deep Learning for Medical Image Analysis demonstrate that eye-tracking data can be used in perceptual experiments and practical applications, i.e., improving diagnostic models by integrating gaze maps with images. This indicates that the benefits of studying gaze extend beyond theoretical understanding to practical implications [22].

## 3. Present Study

### 3.1. ChatGPT and AI Images

In an attempt to identify and name the types of artefacts in AI-generated images, we used the recent literature [23,24], online materials, and guides made by/for fact-checkers and graphic designers [25]. We also thought it would be appropriate to consult the AI itself and thus receive some ad hoc information from the Internet. We used a tool that compiles currently available internet knowledge in a given field: the GPT-4 AI model used in ChatGPT-4o [26]. When asked “How do you recognise AI-generated images?”, it replied, “Recognising images generated by AI algorithms can be difficult, as these images are created by computer models that learn from the data and do not necessarily reflect reality. However, there are some features and techniques that can be used in an attempt to identify such images.” It went on to list some of them:

1. Inconsistency or unreality: AI-generated images are often likely to contain inconsistent or unrealistic elements. For instance, they may feature surreal scenes, impossible perspectives or unusual combinations of objects.
2. Lack of details or overly elaborate particulars: AI images may lack details which are usually found in natural photos or contain overly refined details that do not correspond with the rest of the picture.
3. Repetitiveness or symmetry: Some AI algorithms may introduce repetition or symmetry to their images, which is less common in natural scenes.
4. Generative errors: AI algorithms sometimes make generative errors, which can manifest as artefacts or irregularities in the image. These can include distortions, streaks, cracks, or other unexpected visual effects.
5. Metadata analysis: you can also try to analyse the metadata of the image, such as creation date, author, or source. AI images may have no real metadata or include information that implies they have been automatically generated.
6. Use of image recognition tools, such as content analysis algorithms, to evaluate the content and nature of the image. These tools, however, can be of limited help if the image is unique or difficult to classify.
7. Human expertise: In some cases, it is difficult to know exactly whether an image has been generated by an AI, so human expertise may be necessary for an accurate assessment.”

### 3.2. Objectives and Questions

The study described in this article is the first in a series of planned experiments to determine whether participants can distinguish between authentic images and ones generated by AI and to examine the factors that influence the recognition of various categories of artificial images. Eye-tracking technology was used to this end. As this is the first study in the series, it was also considered a preliminary investigation that might test the proposed research methodology. The sample population for this study consisted of students of Polish origin.

For this reason, the following research questions were formulated:

1. How do users recognise AI-generated images and what factors are crucial to this?

A diverse research group helped answer the question of whether the participants’ initial knowledge of the generative capabilities of the algorithms contributes to the final results.

2. Do differences in the participants’ level of knowledge of generated AI images affect their recognition of the images?
3. Do gender differences influence the ability to recognise AI-generated images?
4. Does age affect participants’ ability to recognise AI-generated images?

It is no accident that ET was chosen as the research method, as it allows the individual perception system of artificial graphics to be investigated. ET measures are non-invasive and ET studies provide insights into users’ cognitive styles while exploring interfaces [27] and performing specific information tasks [28]. In this context, the following question gains relevance:

5. Will the analysis of eye movement add to the observations from the questionnaire and contribute to conclusions about the recognition of artificial images?

Taking into account the initial findings on the subject from ChatGPT, this research verifies its check list and hence verifies whether AI comprehensively communicates how the user perceives images. In parallel, an inverted question can be asked:

Do people really interpret AI images in line with the “rules set” by it (these principles have been listed previously as 1–7)?

## 4. Methods

It was noticed that steady gaze positions, or fixations, associated with overt visual attention are not randomly distributed over a scene consisting of objects [29,30]. As ET research has shown, the gaze path is driven by the observer’s prior knowledge, expectations, strategies, or specific tasks like having to recognise or search for objects within the image [31]. The authors of this study hypothesised that research on AI images in many contexts such as scanning, recognition, and comparison can be enhanced by applying eye gaze methods.

An ET experiment was conducted during June–July 2025 with the GazePoint GP3 HD working at 150 Hz connected to a Dell laptop (Inspiron 5590 i7 RAM 16 GB, graphic card Nvidia RTX 2060) along with a second 27-inch monitor. The distance of the study participant from the monitor and the eye tracker was approximately 70–80 cm as recommended in other studies [32,33]. To prevent possible distractions, the workspace was covered with a folding screen.

**Figure 1:**
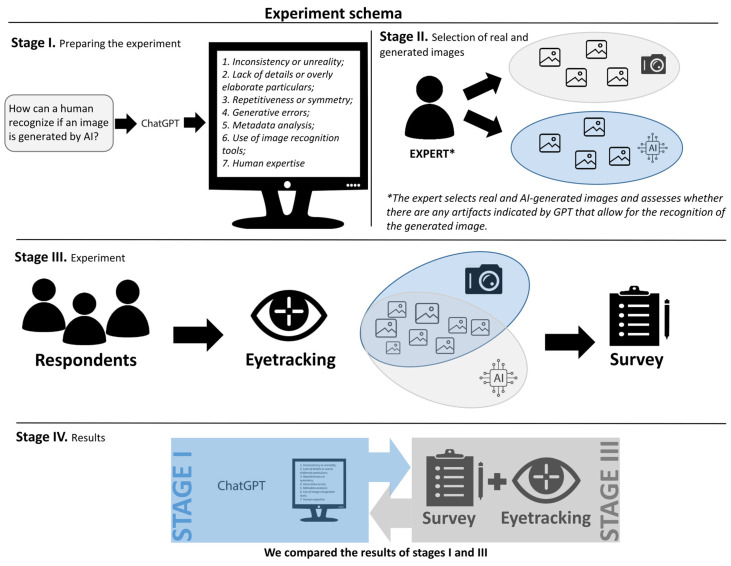
The scheme of experiment’s stages including ChatGPT response analysis (Stage I) and preparing the set of images (stage II).

Given the application requirements, the participants were subjected to a double nine-point calibration. At the beginning of the experiment, the respondents were shown a brief instruction reminding them to avoid unnecessary body and head movements. The participants viewed a series of images displayed for 10 s (Stage III on Figure 1). During the image exposition they had to answer this question: “Is the image real or generated by AI?” The researcher then wrote down the answers on the form.

In order to not disturb the natural eye gaze, the participants were asked about the reasons for their decisions after the session [33]. Then, the respondents were asked to scroll through PowerPoint slides with reduced images used in the experiment and give reasons for their choice. The images were presented in a different order than in the experiment. At this stage, the participants were asked to justify why they believed a particular image to be real or generated by AI.

Data were gathered by Gazepoint Analysis Standard Edition (v6.8.0) software, which also provides visual analysis of both gaze paths and AOI. For selected statistical tests and further inference, XLSTAT 2021.2.2 software including R scripts was used.

### 4.1. Material

In the study, 24 images from six different subject categories were used (see Appendix A): human faces, pets (cats, dogs), landscape, architecture, art, and cars. This categorisation was chosen due to the advancement in GAN algorithms in recent years. These models create high-resolution images that are often indistinguishable from real photographs [4,5], as the authors were aware of thanks to their vast experience in AI art and education. The following initial criteria were adopted for the study:

- Faces: modern AI models are increasingly effective in generating digital representations of human faces. On the one hand, they can be used in marketing or graphic design without needing to navigate sensitive data, copyright, or image rights. At the moment, projects created solely by AI are not protected by copyright [34]. On the other hand, they pose a significant threat when used for manipulation via deepfakes.
- Pets: all AI algorithms used to generate computer graphics struggle to reproduce all the details found in nature. The more complex the object, the greater the likelihood of artefacts. Therefore, it is a serious challenge for AI to accurately reproduce an animal’s fur and movements.
- Architecture: today’s advanced computer graphics, as applied to games and cinematography, are hard to distinguish from the real world. So, for subjects who frequently use such applications, the task of recognising generated photos may prove easier than for the rest of the population.
- Cars: contemporary artistic digital photography and editing play a crucial role in the promotion and marketing of leading automotive brands worldwide. Current generative algorithms can significantly facilitate marketing processes and are also responsible for creating artistic shots and even prototypes whose authenticity of origin can be challenging to assess.
- Landscapes: the generation of realistic landscapes is one of the natural applications of contemporary AI algorithms; however, accurately reproducing the diversity of nature and details such as vegetation patterns, lighting, and atmospheric conditions remains a challenge. AI models perform well in creating general compositions but struggle to maintain consistency with actual geographical conditions.
- Art: images created by AI in the impressionist style and other artistic movements require complex technical skills and an understanding of artistic intent. While AI can formally imitate style, it often lacks the interpretative depth characteristic of human creativity. As a result, these works are among the most challenging to conclusively assess in terms of authenticity and artistic value.

Each category included two genuine photos and two created by AI. The GPT-4o engine was used to generate images of cars, buildings, nature, and architecture. The generation process involved entering detailed descriptions into the OpenAI engine to obtain maximally realistic images. The generation mechanism, except for the “Faces” category, was analogous.

An example prompt used to generate a car image was “*Professional, realistic photograph of a black BMW 5 Series parked in the evening in front of a modern office building in the city centre. The car is illuminated by streetlights, with reflections of city lights visible on the bodywork, detailed grille and headlight features, with glass building facades and subtle street traffic in the background*.”

For the “Faces” category, randomly generated images from the ThisPersonDoesNotExist service were used. Authentic photos for the “Buildings” and “Faces” categories came from Google’s Creative Commons collection, while car photos were taken from the Racecars website (racecars24.co.uk). The most advanced, photorealistic items were selected from several generated pictures, after a discussion; the other category consisted of professional, artistic photographs from private collections.

### 4.2. Subjects

A total of 36 participants (21 women and 15 men) took part in the experiment. The test subjects were adults, aged from 20 to 68 years (Me = 36.5) in a variety of occupations (journalism, graphic design, education, history, computer science). The majority of the participants—21 of the 36 who took part—work (operate, edit or create) with digital images. The participants were also asked about their experience in generating AI images: 15 answered positively and mentioned that they use tools such as ChatGPT, Midjourney, Copilot, and Canva.

## 5. Results

### 5.1. Recognition

During the conducted experiment, a total of 24 colour images were presented to the participants in a random order. In the set of multi-categories 12 images were graphics generated by AI (marked as true—T), while 12 others represented real photographs (false—F) including a face, a cat, and a car. In the overall analysis the participants answered “true” a total of 605 times (70%), while 864 was expected (Table 1).

Having analysed the data concerning the identification of real images according to sex (Appendix A Table A1), it would seem that men generally performed better regarding both AI images and real photographs. However, linear mixed methods tests (mixed-effects models that combine fixed effects and random effects, thus enabling data to be modelled with grouped or repeated measurements) show that sex does not influence recognition significantly: for real and artificial images, *p* = 0.149 and *p* = 0.432, respectively, for a significance level of *α* = 0.05. A Type III test assesses the statistical significance of effect after accounting for the influence of all other variables in the model. Familiarity with digital graphics, especially its generative version, varies across different age groups. To test how age influences the identification of the pictures, the data were categorised according to generations X (45–60), Y (29–44), and Z (18–28) (Appendix A Table A2). It was found that age has significant effect on recognising AI images (*p* = 0.05, *α* = 0.05) and real photographs (*p* = 0.042, *α* = 0.05): Gen Z made the fewest mistakes, Gen X the most.

According to the authors’ expectations, identification of real vs. AI-generated material should be related to practical knowledge about graphic design as well as familiarity with generated images. However, mixed methods tests do not confirm such a connection for most of the combinations. For the first variable—knowledge about digital images (Appendix A Table A3)—*p*= 0.360 and *p*= 0.457 for human and AI images accordingly. However, the second one—operational knowledge about AI graphics (Appendix A Table A4)—was only statistically significant for recognising real photographs (*β* = 1.152, *SE* = 0.531, *t*(38) = 2.168, *p*= 0.036) but not AI images—*p*= 0.726.

Table 2 presents the recognition results for each of the six categories. The lower the factor of recognition, the more advanced the GAN algorithms and vice versa; the higher the factor, the more the algorithms need to be improved. Generated pets (81%) and real photographs of architecture (92%) were the easiest to recognise. Similarly, photographs of cars had a high recognition rate. AI-generated cats and dogs as well as landscapes are still far from photorealistic. However, pictures and photographs of art were indisputably the most difficult to identify. As participants claim, this type of AI image requires initial knowledge about the original artwork. Nonetheless, an expert in fine arts recognised the AI-generated picture not related to any original based on the repetitions of colour gradients in different areas (“Impressionists did not use the same colour transitions in one painting”).

During the subsequent (verification) stage, after displaying the full resolution images, the participants were presented with smaller ones accompanied by questions aimed at understanding the reasons for their choices. During the ET experiment, the participants always managed to answer within the allotted time. However, in the next stage the participants could control the exposure time by clicking the mouse and were able to focus fully on the task at hand. The most common reasons for assessing photos as real were as follows:

(1) For the faces: particulars, lighting, human eyes, natural skin imperfections, mimics, and well-visible shadows.
(2) For the pets: natural surrounding background, natural fur, proportions, cat whiskers, and natural colours.
(3) For the cars: hubcap, normal edges, real rim, reflectance, real reflections, light, no defects, natural shadow play, and foreground.
(4) For the architecture: light reflection and refraction, physics of water particles (fountain), arrangement of objects, and true markings of objects.
(5) For the landscapes: natural colours, natural lines, light reflection, blurring, foreground and background, and intuition.
(6) For the art: surface texture, traces of brush movement, author’s signature, and intuition.

The analysis of answers regarding AI images shows the following observations:

(1) For the faces: sharp details, lighting, reflection in the eyes, outline of pupils, ideal skin, hair, earrings, irregular eyebrows, and visible retouching.
(2) For the pets: smooth fur, unnatural colours, visible retouching, mistakes in the silhouette, and proportions.
(3) For the cars: mistakes in licence plates, logotype, absence of a driver, no motion blurring, foreground–background sharpness, and unnatural light reflections and shadows.
(4) For the architecture: lighting and shadows, no stylistic cohesion, mistakes in architectural details, unnatural silhouette of people, and inconsistencies in the environment.
(5) For the landscapes: enhanced colours, perfectly rendered lines and textures (features as hyperrealism [14]), mistakes in light reflection and blurring, and intuition.
(6) For the art: surface texture, traces of brush movement, author’s signature, and intuition.

One essential contribution to the future development of such research was feedback from the participants at the end of the experiment regarding the difficulty of the assignment rated on a scale from 0 (very easy) to 5 (very difficult). The average score was 3.8. The majority (29 respondents) evaluated the difficulty as 3 or 4 while 7 rated it 4.5 and 5.

### 5.2. Visual Perception

**Figure 2:**
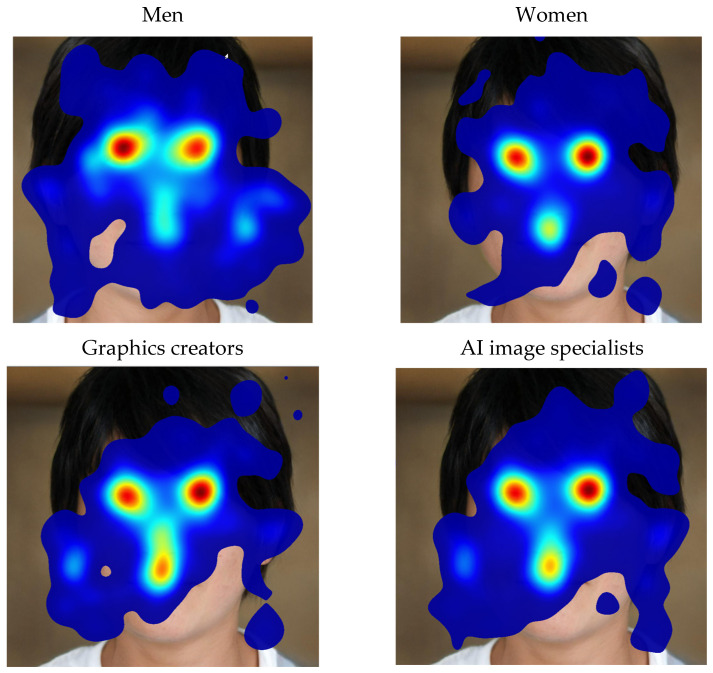
Heatmaps (10 s, 70% size) of AI-generated face for different subject groups. Colors indicate gaze intensity, with red showing areas of highest visual attention and blue showing areas of lowest attention.

We start analysis from images having extreme accuracy rate. The AI-generated face (2, Appendix A) with the lowest value (0.22) was visualised through heatmaps (Figure 2) for various groups of users. The areas around the ears were observed more intently by men and people who work with digital images, because this is a place where potential AI hallucinations may occur. To confirm the differences in perception by selected groups of users, we need to compare statistics within AOIs. Heatmaps give us rough results of users’ perceptual schemes. This tool, in combination with users’ comments during the second phase of the experiment, helps us to draw featured areas of images.

**Figure 3:**
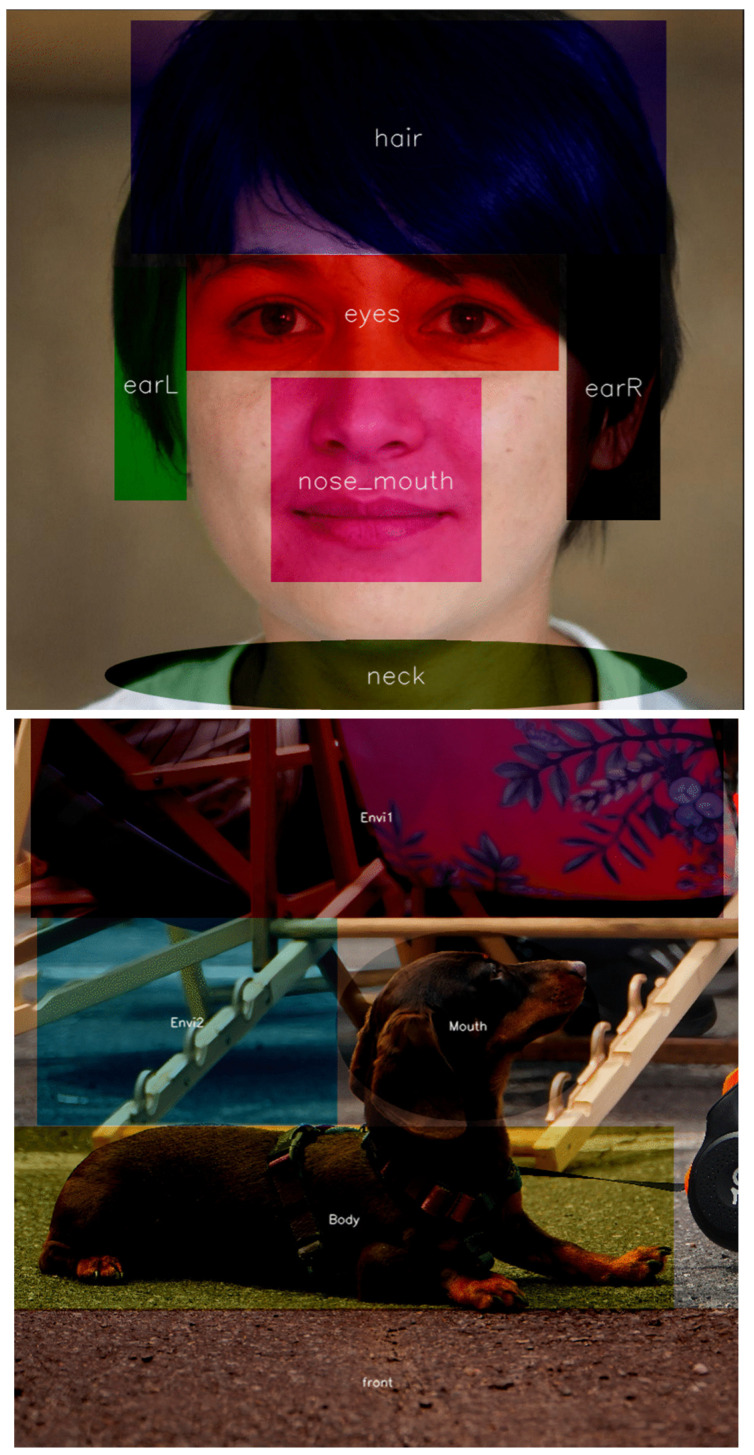
AOI construction for selected images: AI generated face (**top**) and real photograph of pet (**down**).

Therefore, the eyes, nose, ears, hair and the neck were defined as AOI for portraits (Figure 3). Because the subgroups were not equally populated (15 and 21), we focused on relative measures such as viewing time % and average revisits per subgroup.

The muzzle, body, and paws characterised AOIs for pets. In the case of cars, AOIs consisted of a front part featuring the logotype and licence plates, wheels, the silhouette of a driver inside as well as the interior—in particular, the driver’s silhouette—and the environment (the road and background). In landscapes, AOIs were determined by the shoreline, tree line, horizon, clouds, and vegetation in the foreground, whereas in architecture, AOIs included structural lines, with the silhouettes of figures on the street also attracting attention. Visual attention directed to the environment was defined as 100% minus the cumulative share of fixations on foreground objects. For instance, if 45% of overall attention was allocated to the head and 20% to the body, the remaining 35% was assigned to the background. In Table 3 and Table A5 (Appendix A), we can see statistics of the most featured AOIs with an essential difference between groups of users (the largest variances are bold). The main observation is that the largest quantitative differences in visual perception are between men and women. Representatives of other subgroups scored close to the average (Table 3).

### 5.3. Multiscale Analyses

The empirical dataset obtained with a sampling rate of 150 Hz from 36 subjects and 24 images amounted to 531, 231 rows and 51 columns. Fixations data constituted 1.8% of all eye gaze data—thus, the working set was reduced to 10,016 rows. Zero-value columns and rows were then eliminated from the dataset. Four special ET measures connected with fixations (fixation duration and count) and saccades (magnitude and vector between current and last fixations) were selected for further analyses.

**Figure 4:**
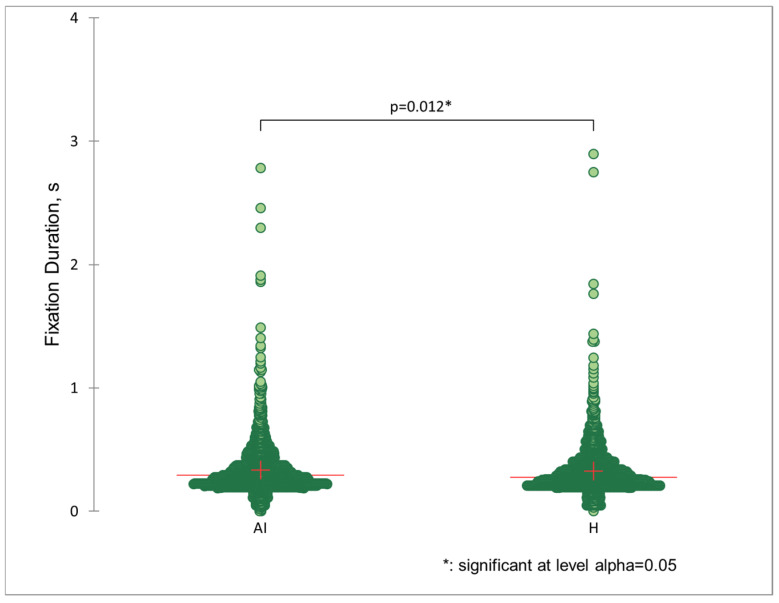
Differences in distribution of fixation duration for AI and human origin images evaluated by *t*-test. Each dot represents single fixation. Scattergrams show the fixations of all participants.

Fixations for AI images and real photographs are balanced in the dataset: 49.68% and 50.32% accordingly. Time-based differences between these samples were evaluated via a *t*-test. The difference between the average fixation duration for AI images (*M*(4976) = 0.33 s, *SE* = 0.003) and human origin pictures (*M*(5039) = 0.31, *SE* = 0.002) is statistically significant: *t* = 1.95, *p* = 0.012, *α* = 0.05 (Figure 4). No significant influence of other measures was observed.

Differences in the perception of images by various groups of respondents was also evaluated by statistical tests. Fixation duration and saccade magnitude were selected as dependent variables. They both have a moderately positive skewed distribution with a skewness (Pearson) of 1.4 and 1.2, respectively. To compare samples when the data distribution is not normal, non-parametric tests are recommended. Variables such as sex, age category and the knowledge of computer graphics and familiarity with AI graphics were considered as explanatory.

Table 4 shows the main statistics of fixations and saccades for men and women. The difference in the average fixation duration and saccade magnitude between these groups assessed through a Mann–Whitney U test is statistically significant: *p* < 0.0001, *α* = 0.05. The men make fewer and longer fixations; their average saccades are shorter than the women’s.

The same Mann–Whitney U test was applied to evaluate the difference in the fixation and saccade parameters for those familiar with computer graphics or AI imaging in relation to the rest. Those with practical knowledge about digital images and AI graphics generated fewer fixations which were longer, while the jumps between them were shorter (Table 5). These differences are statistically significant: *p* < 0.0001, α = 0.05.

According to Kruskal–Wallis test, age and generation of participants (explanatory variable) reveal significant differences in fixations duration (*p* = 0.000, *α* = 0.05). Gen Z generates the least fixations, which are long in duration, while Gen X the most and shortest fixations. (Table 6). Significant differences in saccade magnitude are observed between generations Z and Y (*p* < 0.0001).

## 6. Summary and Discussion

ET heatmaps and AOI effectively illustrate how artefacts were sought in the photos displayed for 10 s. While attention maps derived through Gaussian elimination provided a simplified overview of fixation patterns, this approach resulted in significant information loss, limiting its applicability for in-depth perceptual analysis [35]. However, basic ET metrics such as fixation counts, analysed across participant subgroups defined by sex and expertise, revealed meaningful differences in visual processing. Whereby, experts’ categories include people working with digital images or those working with generative graphics.

AOIs focused on critical facial features—eyes, mouth, nose, ears, and hair patterns—were treated as salient perceptual anchors [29,36,37]. Notably, men and experts exhibited heightened scrutiny of the ear regions, which often harbour subtle AI-related artefacts in hair–skin texture transitions.

Comparable gaze patterns were observed in images of pets, where the participants prioritised the muzzle, ears, and paw regions, the latter serving as cues for realness through implied animal weight. The examination of pet contours further underscored known challenges in rendering fur in computer-generated imagery, with artefacts typically manifesting along fur-background boundaries.

In car images, perceptual attention was directed toward model-identifying elements such as logos, headlights, and mirrors, as well as functional components like wheels, door handles, and the hood. AOI-based statistical analyses substantiated perceptual differences between individuals with varying familiarity with digital and AI-generated graphics. Two fixation-related ET metrics demonstrated significant interactions between stimulus perception and participant expertise; graphic practitioners made longer fixations with shorter transitions, reflecting enhanced visual search efficiency. Graphics practitioners perceive slides longer, making shorter transitions between fixations—they know “how to see” and perform it more effectively. Respondents with advanced knowledge on generative graphics reviewed images in search of artefacts—typical errors for GAN algorithms. So, the first four ChatGPT prompts (cf. Introduction)—unreality, lack of details, errors and repetitiveness, as ways of recognising artificial images by humans—correlate with the given observations. It is worth noting that graphics specialists focused on lighting fidelity reflects. Contemporary designers have substantial knowledge about the propagation of light and photometric phenomena.

Gender-based perceptual differences were also evident, with balanced group compositions bolstering the robustness of these findings. One observation is that objects in the foreground attracts women’s attention. They noticeably interact more with nature, beauty (sky, water), and emotional accents (eyes, muzzle). However, men tend to spend longer scanning the environment where the objects are located. The exception are cars, which disturb men’s analysis of the background.

Using ET, it was not possible to observe subtle cognitive strategies [23], such as comparing foreground and background focus or symmetry analysis, which advanced respondents performed automatically. Neuroactivity measurements may explain more profound cognitive–perceptual mechanisms, which the authors plan to investigate by integrating different recording techniques. However, ET metrics supported by statistical tests helped detect differences in the visual perception between men and women, and graphics experts in relation to others.

In one of the compared subgroups, participants exhibited fewer but longer-lasting fixations than others, while their saccades were shorter. This pattern of perceptual behaviour characterises both digital graphics and AI image experts, as well as Generation Z and men (see Table 4, Table 5 and Table 6). Assuming that experts make the best use of their knowledge in tasks involving the evaluation of AI-generated images, Generation Z appears to be acquiring similar competencies. Given the uneven distribution of age group members in the current sample, these results should be confirmed on a more representative group. However, recent studies report that visual perception strategies used to judge image authenticity vary clearly across age groups [21,22,38]. Younger users are generally faster at detecting critical details that help identify image sources.

Men in this study showed visual perception patterns—such as fixation duration and saccade length—closely resembling those of recognised digital and AI image experts. This similarity suggests that men may either have greater familiarity with digital and AI-generated images or use more expert-like strategies when evaluating image authenticity. This conclusion is supported by the fact that the expert group was gender balanced. Consequently, men’s gaze behaviour aligns them with the expert group.

The increased fixation duration when perceiving AI-generated images may be explained by the fact that respondents often noted the presence of artefacts in images generated by artificial intelligence in their answers. While the number of fixations correlates with the information-search process, fixation duration should be interpreted as reflecting the engagement of cognitive processes such as reasoning and memory to assess whether a given artefact could appear in a real image. It is important to emphasise that AI-based systems exhibit numerous errors related to the depiction of depth of field across different planes, difficulties in blending detailed textures—for example, at the boundary between the ear and hair—as well as logical errors such as asymmetrical arrangements of tiles or floorboards.

Nonetheless, these observations need to be verified with a larger sample of participants to ensure robustness. Nevertheless, there were over-interpretations in the subjects’ evaluations: features that, to some people, seemed to be evidence of a genuine photo, to others were indicative of AI creativity. Such behaviour is inherent in this type of experimentation toward AI image recognition [37]. It can be presumed that this type of research will be accompanied by overconfidence and excessive suspicion unless users expand their practical knowledge of the reproducibility capabilities of AI algorithms which, after all, are constantly evolving. In this regard, further research into developing techniques for identifying the authenticity of images and education in detecting digital manipulation may provide the key to effectively dealing with the challenges posed by the development of AI in image generation.

## 7. Conclusions

The study was designed to assess the authenticity of pictures generated by AI by comparing them with real photos. Six categories of objects were included in the analysis: faces, pets, cars, architecture, landscapes, and art. Generated pet images and real photographs of architecture were the easiest categories to identify. These categories are still far from photorealistic and the human eye could grasp the essential details. The art category proved the most difficult for identifying real vs. artificial, because initial knowledge about the original artwork is required.

The majority of the respondents correctly assessed the authentic photos, pointing out details like natural lighting and visible imperfections as evidence of their authenticity. Their comments connected with artefacts were largely consistent with the ChatGPT prompts. However, AI algorithms can underestimate experts’ knowledge of physical phenomena such as illumination. Importantly, the largest quantitative differences in visual perception occurred between men and women. Women’ visual attention focused on foreground elements and emotional scenes, in particular, while the men tended to concentrate more on the environment.

The main conclusions based on ET measurement are the following: (1) AI images are analysed with fixations about 20 ms longer than real photos; (2) practical knowledge about digital images meant that such respondents presented fewer fixations that were longer on average than those of the rest of the group. These differences are statistically significant. Perhaps AI pictures contain some unknown structural features (smoothing, edge sharpness, borders of texture) that draw the respondents’ attention regardless of their level of familiarity.

Taking into consideration the growth of popularity of GAN products among young users (some of the authors conduct classes on graphic design and Human Computer Interaction), this research may well prove valuable in terms of testing the methods used and reveal new problems in AI-oriented communication and education.

### Recommendations for AI Developers and End-Users

For AI developers, it is important to recognise two complementary processes. On the one hand, users demand higher quality in generated images, which requires improving the accurate rendering of elements such as human faces, logos, and architectural details. On the other hand, the increasing realism of synthetic content raises the urgency of advancing tools that can reliably detect whether an image is authentic or AI-generated. Future work should therefore not only strive for more photorealistic generation but also ensure that equally robust detection mechanisms are developed in parallel to balance innovation with safeguards against misuse.

## Figures and Tables

**Table 1:** Number of recognised AI-generated pictures (T) and real photographs (F).

	Recognised	Expected
T	274	432
F	331	432
**Total**	**605**	**864**

**Table 2:** Recognition rate by image category.

Accuracy	Architecture	Landscape	Cars	Faces	Pets	Art
AI images	0.53	0.81	0.63	0.5	0.81	0.58
photographs	0.92	0.61	0.78	0.68	0.56	0.61
**Total**	**0.72**	**0.71**	**0.70**	**0.68**	**0.68**	**0.59**

**Table 3:** The featured AOI statistics for different groups of users (gc—graphics creators, AI is—AI image specialists).

	AI Images					
AOI		Viewing Time %				
		Men	Women	Gc	AI is	All Users
face2 *	ears	**15**	**7**	11	13	10.7
	mouth and nose	**14**	18	19	**20**	16.4
	hair	**13**	10	**9**	9	11.6
	eyes	**35**	**40**	37	37	37.9
pets2	background	**22**	**11**	15.7	11	15
car1	road	**4**	**16**	12.6	12	10
	**Human origin images**					
AOI		Viewing time %				
		Men	Women	gc	AI is	**All users**
face2	face	45	49.6	45.4	47.7	47.6
	scarf	**10.5**	**5.2**	7.9	6.0	7.2
	buttle	**10.5**	**18.5**	13.8	17.4	15.4
pets1	cats	**32**	**42**	38	39	36.8
pets2 *	muzzle	**13**	**20**	18	**20**	16.9
	chair	**35**	**25**	29	24	29.3
archi2	laundry	**4**	**8**	6.1	7	6.8
	aircon	**14**	**10**	12.4	12	11.5
landsc2	sky	**9**	**14**	9.2	11	11.3
	water	**11**	**14**	11.2	11	13
cars1	front	**4**	9	6	**10**	7.4
	car	**62**	**54**	57	59	57.1

* On Figure 3.

**Table 4:** Statistics of fixations and saccades according to the sex of the participants (Fix #—fixations number).

Sex	Frequencies	Fix #/ Person	Fixations Duration		Saccade Magnitude	
			Mean	Standard Error	Mean	Standard Error
Female	5495	309.0	0.318	0.003	526.6	6.2
Male	4507	300.5	0.335	0.003	482.0	6.8

**Table 5:** Statistics of saccade magnitudes according to familiarity with digital graphics and AI images (Fix #—fixations number, gc—graphics creators, AI is—AI image specialists).

Experience		Frequencies	Fix #/ Person	Fixations Duration		Saccade Magnitude	
				Mean	Standard Error	Mean	Standard Error
Gc	Yes	6843	210.6	0.335	0.003	497.2	5.4
	No	3153	325.6	0.315	0.002	526.7	8.6
AI is	Yes	3462	230.8	0.334	0.002	490.7	7.9
	No	6540	311.4	0.326	0.004	514.9	5.9

**Table 6:** Statistics of fixations and saccades according to age and generation (Fix #—fixations number).

Age Category	Frequencies	Fix#/ Person	Fixations Duration		Saccade Magnitude	
			Mean	Standard Error	Mean	Standard Error
X	2450	310.6	0.314	0.002	511.2	7.5
Y	3094	314.2	0.320	0.001	484.1	3.9
Z	4458	292.4	0.337	0.002	515.1	5.1

**Figure A1:**
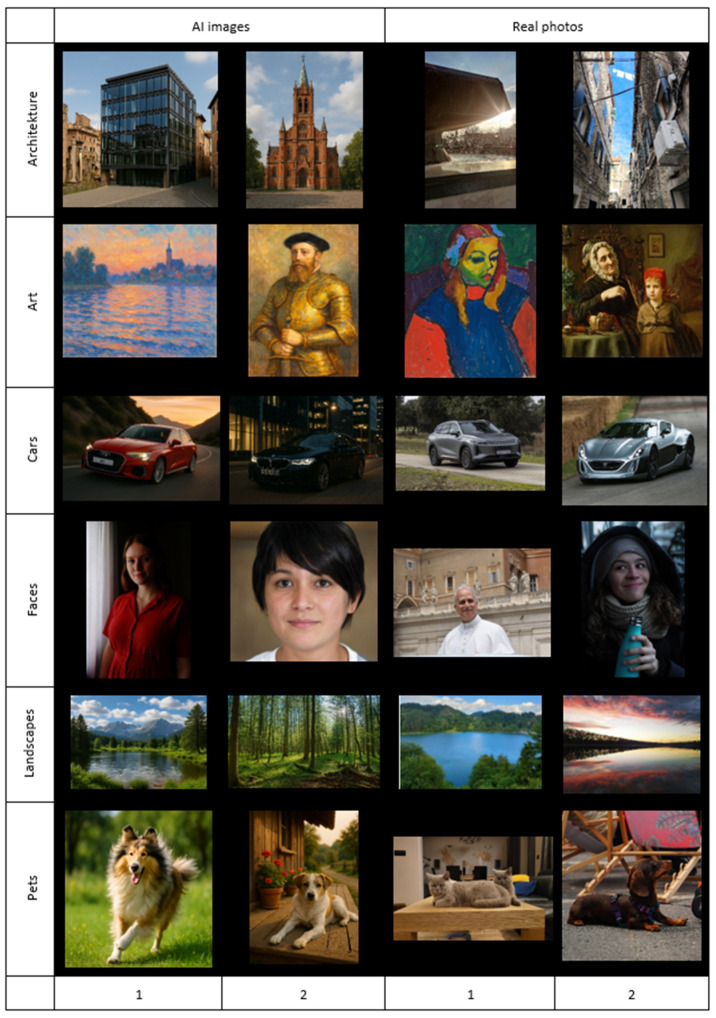
Images dataset used in the study (full size images are available at: https://bitscope.umk.pl/en/images (23 October 2025).

## Data Availability

The data that support the findings of this study are openly available in Zenodo at: https://zenodo.org/records/17479248 (23 October 2025).

## Appendix A

**Table A1:** Number of recognised and AI-generated pictures (T) and real photographs (F) by females and males.

	Recognised		Expected	
	F	M	F	M
T	155	119	252	180
F	186	145	252	180
**Total**	**341**	**264**	**504**	**360**

**Table A2:** Number of subjects according to age category.

Categories	Frequencies	
Gen Z	13	36,111
Gen Y	16	44,444
Gen X	7	19,444

**Table A3:** Number of recognised and AI-generated pictures (T) and real photographs (F) according to experience (E) with digital images.

	Recognised		Expected	
	E	N	E	N
T	107	167	252	180
F	202	129	252	180
**Total**	**309**	**296**	**504**	**360**

**Table A4:** Number of recognised and AI-generated pictures (T) and real photographs (F) according to experience (E) with AI images or no experience (N).

	Recognised		Expected	
	E	N	E	N
T	112	162	180	252
F	148	183	180	252
**Total**	**260**	**345**	**360**	**504**

**Table A5:** Additional AOI statistics for different group of users (gc—graphics creators, AI is—AI image specialists).

AI Images						
AOI		Average Revisits Number				
		Men	Women	Gc	AI Is	All Users
face2 *****	ears	4.6	3.6	4.6	4.4	4.1
	mouth and nose	2.9	4.6	3.8	4.2	3.9
	hair	2.7	2.1	1.9	1.7	2.4
	eyes	4.5	5.1	4.9	4.8	4.8
pets2	background	4.2	2.9	4.8	2.6	3.4
car1	road	**1**	**3.7**	4.6	3.3	3.4
**Human origin images**						
**AOI**		**Average revisits number**				
		**Men**	**Women**	**gc**	**AI is**	**All users**
face2	face	4.2	3.5	3.8	3.8	3.8
	scarf	**2.8**	**1.8**	2.3	2.2	2.2
	buttle	1.6	1.8	1.8	1.7	1.7
pets1	cats	6.7	7.7	6.8	7.1	7.3
pets2 *****	muzzle	3.1	3.9	3.2	3.6	3.5
	chair	4.1	4.6	5.3	4.7	5.3
archi2	laundry	2.3	2.2	1.9	2.2	2.2
	aircon	2.4	1.6	1.7	1.7	1.9
landsc2	sky	2.3	2.8	2.3	2.3	2.7
	water	2.4	2.3	2.4	2.0	2.4
cars1	front	1	1.3	1.2	1.2	1.3
	car	3.8	3.9	3.5	3.5	3.9

* On Figure 3.
